# Observations and Reflections on the Disease Produced by the Action of the Canine Virus on the Human Body

**Published:** 1819-11

**Authors:** James Mease

**Affiliations:** Philadelphia.


FOR THE LONDON MEDICAL AND PHYSICAL JOURNAL.

***Observations and Reflections on the Disease produced by the Action of the Canine Virus on the Human Body.*** **By JAMES MEASE, M.D. Philadelphia.***

THE disease produced by the action of the virus of a rabid animal of the genera canis and felis, may be justly styled the *opprobrium medicorum;* for, notwithstanding the length of time that it has been known, the numerous works written upon it, and the various modes of treatment pursued, we every year see or hear of instances of death from it. So long, therefore, as it continues to baffle the skill of physicians, it is worthy of investigation; and the advice which I gave many years since on the subject, may with propriety be repeated: that is, to publish the history of every case and treatment, whether successful or the contrary, in order that we may adopt the remedies by which it was cured, or avoid those that failed. In the United States the disease frequently appears; but has as yet never been cured. During the past winter, one case happened in Philadelphia; two years since, one occurred in Frankford, near Philadelphia; two or three in New England; last year, four in Richmond, Virginia. One case also occurred in Montreal; and all unhappily with the same result.

The cases in Richmond and Montreal were particularly interesting to me, because, in one of the former, the preventive treat-

---

* We refer our readers to the eighth volume of our Journal for an account of the chief of the original opinions of this zealous and intelligent physician on this subject. The present memoir will furnish our readers with an account of the history of hydrophobia, and its treatment, in the United States, up to the existing period.—EDIT.

ment was adopted which I many years since showed had so often failed that it was not to be trusted; and the curative remedy that I have long since reprobated, was followed, in the latter, with the same result which attended its use in almost every instance on record in which it had been tried. The reader will at once refer my exception to those cases cured in Calcutta by bleeding. Those in Richmond were doubly important, on account of the total failure of that disgraceful imposition, the Chinese snake-stone, which I have noticed in Coxe's *Medical Museum*, vol. v.

Abstracts of three cases that occurred in Richmond, Virginia, from *Rice's Magazine*, Richmond.

1. Edward Taylor, aged 12 years, was bitten on the 27th March, 1818, in the back of the hand, and in the palm next the thumb, by a dog of the family, which had shown no symptom of madness, except an unusual degree of ill-nature. The dog was soon tied, eat freely, and recognized the members of the family, giving the usual indications of affection when kindly called. The wound was well washed in brine; the snake-stone was afterwards applied four or five times to the wounds, and was said to have performed its office perfectly well. The wound was also kept open for several weeks, and then healed.

On the forty-second day after the bite the boy began to complain. He was purged with calomel and Glauber's salts. In attempting to swallow the solution of the latter, much difficulty was discerned, and he sighed continually. In drinking, the difficulty was not in swallowing, but in getting the water into the mouth: the lips then closed upon it. That once accomplished, the swallowing was perfectly easy. He made powerful efforts to resist the spasmodic motions produced by every attempt to receive liquids; complained of the "beating of his heart;" heat considerable; pulse extremely rapid, but feeble. One hundred drops of laudanum, given in the course of a few hours, increased the restlessness, and aggravated the symptoms. He was perfectly sensible, and his naturally affectionate disposition was increased towards his mother and relations. His eyes became so brilliant as to require an effort to look him in the face. The symptoms became worse rapidly, and in twenty hours from the first appearance of hydrophobia, his agony was over.

2. The second case is related by Dr. Henning, in the *Richmond Inquirer*, July 5.

James S. West, aged two years, was bitten on the 22d May, 1818, through the palm of the hand. The snake-stone was applied, and stuck for some time: it was applied a second time. Lunar caustic was afterwards applied, and also warm poultices; mercurial frictions were used, and calomel given. The next day the eschar produced by the caustic was removed, the wound dilated, and the caustic again freely applied: the wound was dressed with blistering ointment, and a poultice applied over it. By this treatment, the wound discharged freely for several weeks. A slight mercurial action was also produced; when the mercury was laid aside, the wound filled up, and soon after healed.

The disease appeared on the 22d June in the night. The symptoms were, starting in sleep, screaming, and pain in the head; agitation at the sight of, and buzzing of, flies; or by exposure to a current of air, or the reflection of his own image from a looking-glass, (as usual.) An injection of fifteen drops of tincture of opium was prescribed, and directed to be repeated until some effect was produced. Cold applications were directed for the head. The wound was again cauterized, and five drops of laudanum given every half-hour. Seventy-five drops had been taken by early the next morning without any good effect, not even sleep. Perspiration flowed freely, and a frothy saliva issued from the mouth. The jugular vein was then opened; but, before four ounces were drawn, faintness ensued, and the blood was stopped. Between two and three o'clock P.M. the child died.

3. Abstract of a third case of the disease that occurred in Richmond, Virginia, in 1818; related by Dr. TRENT. (*Richmond Inquirer*, Nov. 24.)

The dog discovered no other symptom of the disease than drooping and the refusal of food. The same dog, October 23d, bit a negro man in the naked fore-arm: the dog was confined, and yet fondled on another negro man, Burwell, who approached him, submitted to be chained, and wished to follow him out. While caressing the man, the dog took the man's hand gently into his mouth, but without the slightest disposition to bite: in retracting his hand suddenly, he scratched his fore-finger against a tooth. The dog died in forty-eight hours after the first appearance of indisposition, without discovering any other symptoms of madness. Slight indisposition took place on the 13th November; but on Monday evening, the 16th November, he was seized with a chill, high fever, and great thirst. The next day he complained of shortness of breath, pain in his left shoulder, slight head-ach, and lassitude; had no aversion to fluids; no pain in the wounded finger, which had healed soon after the injury; pulse preternaturally slow, soft and feeble; skin cool; tongue furred. Took an emetic, which he drank with ease, and worked it off with plentiful draughts of warm water: it opened his bowels twice. In the evening, feeling hungry, he attempted to drink some soup, but was seized with a spasm that threatened suffocation. The next day his skin was cool, pulse irregularly frequent and slow; felt shortness of breath, and soreness from his throat to the pit of the stomach; surveyed his face in a glass with composure; drinking soon excited a violent spasm. Two abortive attempts were made to bleed: took fifteen grains of calomel, and thirty of jalap, in pills, with ease; blister applied along the whole course of the spine, another from his throat to the pit of the stomach. More abortive attempts to bleed in the arm, in the two jugulars, the veins on the feet and ankles, and two temporal arteries: six ounces only were obtained; blood thick and black, and never separated. Skin too cool; blisters were applied to his fore-arms and legs, and bottles, filled with hot water, to different parts of his body. In the evening lunar caustic was applied to the surface exposed by detaching two and a half inches of blistered cuticle on the spine of the neck, the pain from which excited several fits of convulsive agitation; swallowed with ease ten grains of calomel and three of ipecacuanha in two pills; took a second dose, which produced free perspiration, nausea, and vomiting. The blisters produced stranguary. He asked several times for drink, but took it only twice, as it excited convulsive agitations. The windpipe was found morbidly dry; the stomach exhibited strong marks of inflammation; diaphragm very red. Dr. Trent remarks, that, except the "inability to make a long breath, there was not a symptom which justified blood-letting;" but supposes, that if he "had been seen on Monday night, blood-letting to fainting might possibly have saved him. Before the case was recognized as hydrophobia, the action of the heart and arteries was prostrated by the impress of the disease on the function of respiration, upon which their action depends." He was sensible to the last, and showed not the least disposition to do injury to any one.

The foregoing cases admit of several deductions, which it may be useful to state; and I notice them with the more satisfaction, not so much because they confirm some of the opinions and positions I first advanced in my Inaugural Dissertation in 1792, and since, in other papers, on the disease, but because they will illustrate the pathology of the disease, dispel error, and, I hope, thus lead to the cure of this hitherto almost unmanageable disease.

1. They show that the stage of the dog's madness has no influence in expediting, or protracting, the operation of the rabid virus on the human body. The dogs that bit the subjects of all the cases in Richmond, were evidently in the very earliest stages of the disease, as appeared by the inoffensive and friendly conduct of two of them; and, in the case related by Dr. Henning, it is stated, that the dog bit the hand of the child, which he put out through the palings of the yard as the dog passed in the street. Having bitten other dogs and animals, "he was pursued with an intention to kill him; but, taking refuge in the house to which he belonged, he was confined, and, before he was killed, evinced unequivocal symptoms of hydrophobia." Now, dogs in the advanced stages of their disease, always run away from home, if not confined. It was formerly supposed, that, the longer the disease had continued in a rabid animal, the more virulent was the poison contained in the saliva.

2. They prove satisfactorily, that the size of the wound has no influence upon the quick or tardy operation of the virus. It was formerly supposed, that, the more virus was infused in the system by the laceration of the dog's teeth, the more sudden would be the attack. The analogies of the small-pox virus, and of that of syphilis, might have prevented the formation of such an opinion, and of its adoption; but they were overlooked. Numerous facts now show its erroneous nature. In my remarks on the case of the boy Yorke, related by Dr. PHYSICK, which I closely attended to, I have adduced several cases to prove the accuracy of the above deductions. (See *New-York Medical Repository*, vol. v. p. 292.) Yorke himself was one.

3. The third case related, shows also how fallacious all preventive remedies must be. The dog bit a negro man in the naked fore-arm, and as yet has remained well; whereas, a slight scratch on the fore-finger by the dog's tooth, infused the virus that took effect in Burwell. Had the man first bitten applied the Gloucester (Virginia) infallible, the Chinese snake-stone *(risum teneatis medici)*, or taken internally any other reputed specific, a new proof would have been adduced of their salutary power in securing the system against an attack of the disease.

4. The foregoing cases, and many more, recorded by myself and others, prove the total inefficacy of the common preventive treatment by the application of caustics to the bitten part. Indeed, after the mass of evidence extant on this head, it is surprising that physicians should torture their patients with them, or place any reliance upon their capability of preventing the operation of the poison. At an early date of my attention to the disease, I was satisfied of their utter inutility, and decidedly expressed myself to that effect, in my inaugural dissertation. The subsequent publication of numerous cases, in which they have been used, under every circumstance favourable to their efficacy, viz. of speedy and severe application after the bite, have served to strengthen my belief in their inefficacy, and urge me to caution practitioners against placing the smallest reliance upon them. Dr. Henning gave them the fairest trial possible, by dilating the wound, and by their severe application, but without success, although the "free suppuration" caused by them was kept up "for several weeks." Mr. GILMAN, of

London, in his Prize Essay on this disease, has given a satifactory explanation of the cause of their failure. "When a caustic is applied," he says, "a new compound is formed,—a saponaceous mass or eschar, which is generally suffered to remain until it sloughs away. Of what, then, is this new compound formed, but of dead animal matter, a caustic, and of a peculiar poison, which we believe to be the cause of hydrophobia? It is true, the neighbouring absorbents are destroyed, so far as the action of the caustic extends; but the canine virus is as likely to extend with it, being only in a state of union from the commencement of this operation, which is continued till the poison is uniformly dispersed through the whole of the adjacent parts, forming an animal soap by their commixture. Hence, by such means, a more extended surface is exposed to the action of the absorbents, which are rendered highly irritable and more active; and in consequence, perhaps, the case becomes more desperate." This is true philosophy. Mr. G. has collected, in one view, a number of recent cases, in which caustics of every description were applied to bitten parts, and in the most severe manner, without preventing the disease. These cases, with Dr. Henning's, ought to satisfy every one that it is torturing, without benefit, to apply them. But many more, that might be added, are quite a balance for those in which Dr. Mosely says he has used them with success.*

* Press of other matter obliges us to defer the remainder of this article to the next Number.—EDIT.

